# Steered Molecular Dynamics Simulations Study on FABP4 Inhibitors

**DOI:** 10.3390/molecules28062731

**Published:** 2023-03-17

**Authors:** Rosario Tomarchio, Vincenzo Patamia, Chiara Zagni, Letizia Crocetti, Agostino Cilibrizzi, Giuseppe Floresta, Antonio Rescifina

**Affiliations:** 1 Department of Drug and Health Sciences, University of Catania, Viale A. Doria 6, 95125 Catania, Italy; rosario.tomarchioct@gmail.it (R.T.); vincenzo.patamia@unict.it (V.P.); chiara.zagni@unict.it (C.Z.); 2 Department Neurofarba, Pharmaceutical and Nutraceutical Section, via Ugo Schiff 6, 50019 Sesto Fiorentino, Italy; letizia.crocetti@unifi.it; 3 Institute of Pharmaceutical Science, King’s College London, Stamford Street, London SE1 9NH, UK; agostino.cilibrizzi@kcl.ac.uk; 4 Centre for Therapeutic Innovation, University of Bath, Bath BA2 7AY, UK

**Keywords:** fatty acid binding protein, FABP4, FABP4 inhibitors, computer-aided drug design, molecular modeling, steered molecular dynamics, drug design

## Abstract

Ordinary small molecule de novo drug design is time-consuming and expensive. Recently, computational tools were employed and proved their efficacy in accelerating the overall drug design process. Molecular dynamics (MD) simulations and a derivative of MD, steered molecular dynamics (SMD), turned out to be promising rational drug design tools. In this paper, we report the first application of SMD to evaluate the binding properties of small molecules toward FABP4, considering our recent interest in inhibiting fatty acid binding protein 4 (FABP4). FABP4 inhibitors (FABP4is) are small molecules of therapeutic interest, and ongoing clinical studies indicate that they are promising for treating cancer and other diseases such as metabolic syndrome and diabetes.

## 1. Introduction

Fatty acids (FAs) are organic compounds characterized by a long carbon chain and a carboxylic acid functional group responsible for several functions in human physiology [1,2]. The chronically elevated concentration of FAs in plasma is correlated to human disorders [3,4] such as diabetes [5], atherosclerosis [6], and obesity [7]. FAs’ high lipophilicity is responsible for their low water solubility; thus, their trafficking requires specialized proteins such as fatty acid binding proteins (FABPs) [8]. Based on their localization, FABPs are classified into different families; FABP4 (aP2 or A-FABP) is the subtype mainly produced in the adipocytes [9]. The research on FABP4 inhibitors started when it was reported that knockout animal models of FABP4 naturally developed protective effects against insulin resistance [10] and other events, such as metabolic syndrome and atherosclerosis [11,12,13]. Indeed, pharmacological approaches with small molecules of FABP4is demonstrated similar results in the phenotype of FABP4-deficient mice [14].

This protein family also has a role in cancer progression [15]. In fact, renal cell carcinoma, bladder, prostate, and other cancer cells [16,17,18] were reported with a non-physiological expression of FABPs. The classical FABP4i, BMS309403, was reported to weaken the migration and invasion of colon cancer cells. These results highlighted the tendency of FABP4 to promote colon cancer metastasis and invasion [19]. It is known that FABP4 leads to abnormal metastasis and aggression in ovarian cancer, contributing to poor prognosis for this [20] and other types of cancers, such as glioblastoma [21].

All of these recent findings suggest that FABP4 targeting may represent a valid and promising therapeutic strategy against oncological conditions.

Various effective FABP4is were developed recently, but unfortunately, none are currently in the clinical research phase [14,22]. Computer-aided drug design is promising in drug discovery and an effective tool for identifying molecular hits such as FABP4is [23,24,25,26,27,28]. In line with our recent interest in developing new antitumor compounds and identifying novel bioactive heterocycles [29,30,31,32,33,34], we herein report the first application of SMD to evaluate the binding properties of FABP4is to the targeted protein.

MD simulations of proteins at the atomic level are a well-established method for describing the behavior of proteins and the protein-ligand interactions involved in cell signaling for disease processes. A special kind of MD simulation is the SMD simulation. With this method, a protein or molecule can be given a directing vector as part of the SMD simulation to examine how it reacts to outside stimuli [29]. SMD simulations, which provide atomic-level resolution of force-probe events, proved to be essential supplements to current experimental techniques [30]. In a typical SMD simulation, one terminus of the molecule is subjected to an external vector while the opposing terminus is fixed in space. This allows researchers to examine how the molecule reacts to mechanical stress and how it can clarify the structure-function relationship of a macromolecular complex that involves either protein-ligand or protein-protein interactions [31,32]. SMD simulations for protein-ligand complexes may be used to determine stabilizing interactions, which help inform the development of drugs that can quickly and readily bind to the active binding site to increase or decrease the activity of the desired protein. SMD was developed to address simple questions from protein conformational changes relative to the study of relevant residues involved in particular interactions. Recently, it was also used to evaluate drug-binding affinity for drug screening. The unbinding force obtained from receptor-ligand dissociation and SMD simulations can be used to estimate a ligand’s binding affinity with reasonable accuracy [33,34,35].

The use of computational simulations proved very advantageous to the pharmaceutical industry, where high precision, low cost, and reduced time and labor in drug development are all priorities. The molecular mechanics Poisson–Boltzmann solvent accessible surface area (MM/PBSA) method is one of the most extensively used computational approaches for calculating the binding affinity, i.e., the free energy of binding (Δ*G*_bind_), between substrate and protein. It was also proven that the MM/PBSA and SMD have a strong connection in assessing the binding affinity of small molecules to a protein [36]. For example, recently, SMD simulations were used to evaluate the binding affinity of antiviral compounds [37,38], studies which also correlated simulation results and MM/PBSA data. These simulations were also used to evaluate molecules for the treatment of cancer-specific inhibitors of allosteric BcrAbl fusion protein for the treatment of chronic myeloid leukemia [39] and histone deacetylases (HDAC) [40].

All of these studies demonstrate that SMD is a powerful tool for predicting the mechanisms of efficient drug binding and a potent hypothesis-generating tool for screening new drug candidates. Due to our recent interest in the design of small molecules able to inhibit FABP4 by using structure- and ligand-based computational tools, we herein report the first application of SMD to evaluate the binding affinity of small molecules as inhibitors of FABP4. The aim of this study was to optimize a simple method to employ SMD for the fast evaluation of novel small FABP4is molecules.

## 2. Results

The ligand binding sites of FABPs, shown in X-ray crystallographic studies, have been known for decades. However, the mechanism of how the ligands access their binding sites remains challenging to identify, and computational SMD proved the existence of basically one ligand escaping its route despite identifying at least two possible portals. In all successful dissociations, the palmitate molecule was established to come out from essentially the same region [41]. The same area was used in our study to dissociate the selected ligands.

**Figure 1:**
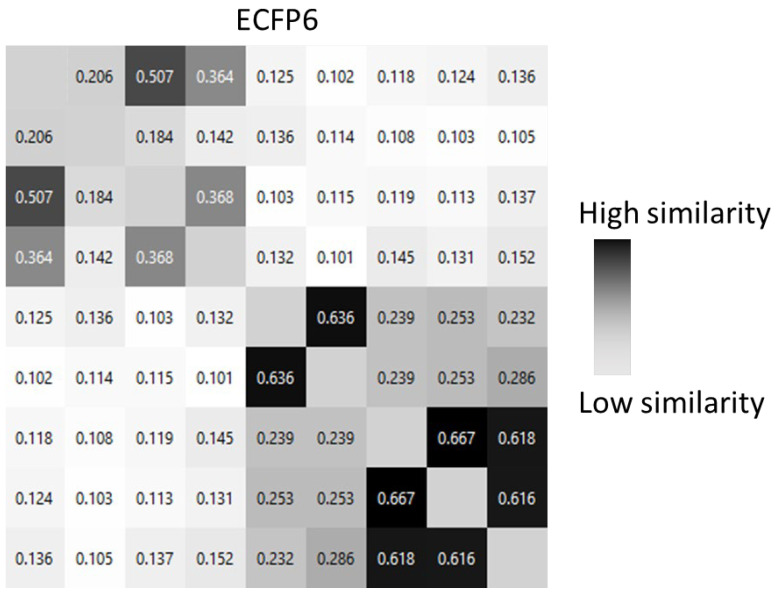
Similarity matrix calculated with ECFP 6 for molecules **1**–**9**.

The key component for running the SMD simulation is the initial configuration of the system. The Jarzynski equation requires configurations sampled from an equilibrium distribution with *λ* = *λ*_0_. These configurations can be obtained in two ways: through a Monte Carlo simulation or by periodically saving snapshots from a molecular dynamics simulation of the system with the reaction coordinate constrained to the desired initial value. The second option was used in this work. The two key parameters fundamental to interpreting the reaction unfolding are the acceleration estimated in pm/ps^2^ and the distance measured in Å. These settings allowed us to calculate and obtain the total energy measured in kcal/mol required for the ligand to travel to a previously set distance. Structural heterogeneity was ensured by selecting different molecules as a measurement of pairwise similarity calculated by using circular fingerprints (Figure 1). Extended-connectivity fingerprints (ECFP) are circular topological fingerprints optimized and designed for molecular characterization, similarity searching, and ligand-based molecular modeling. They are among the most common and best-performing similarity search tools in drug discovery, and they are used in many applications [42,43]. The similarity matrix from the ECFP evaluation was also compared with the Tanimoto similarity index, and a similar pattern was also identified in the Tanimoto matrix (Figure S1, Supplementary Materials), confirming the good dissimilarity between the chosen compounds.

Table 1 reports the p*K*_i_ values obtained among the various molecule sets between the calculated energies of binding by docking scoring functions, the SMD simulations’ calculated total energies and F_max_, and their experimental inhibition constants.

**Figure 2:**
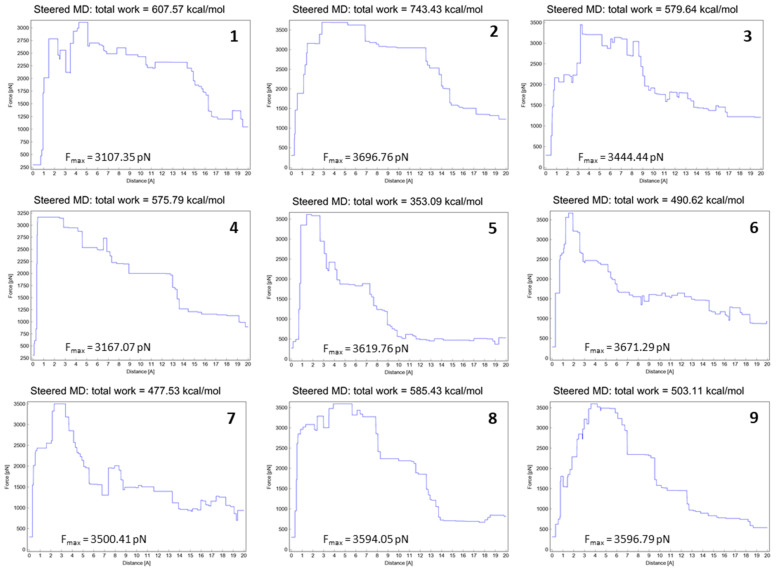
Total energies and F_max_ from SMD experiment (20 Å) for molecules **1**–**9**.

Figure 2 states the total energies and the max force (F_max_) obtained from each SMD experiment. Simulations were carried out in triplicate with double-blind conditions using different hardware systems to ensure the homogeneity and repeatability of the experiment.

**Figure 3:**
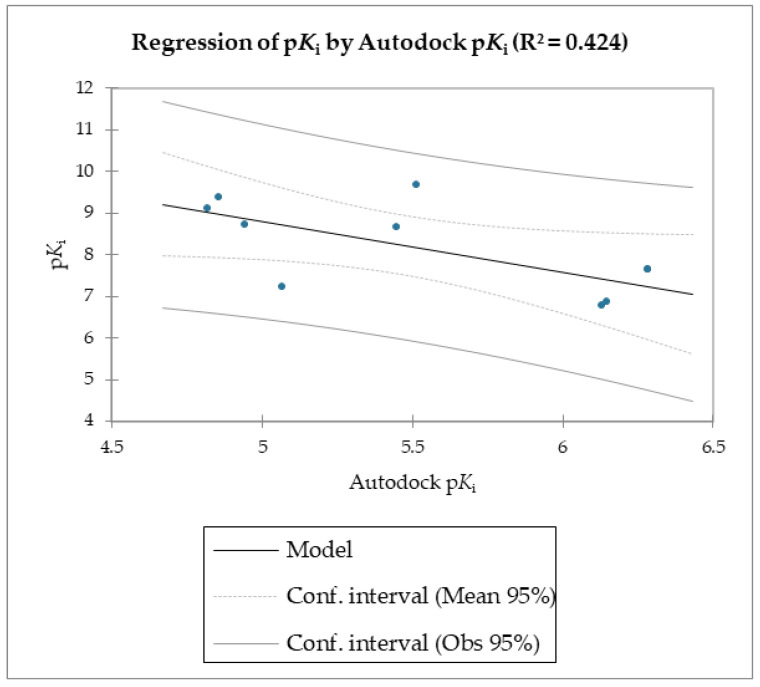
Regression of p*K*_i_ with Autodock-calculated p*K*_i_.

**Figure 4:**
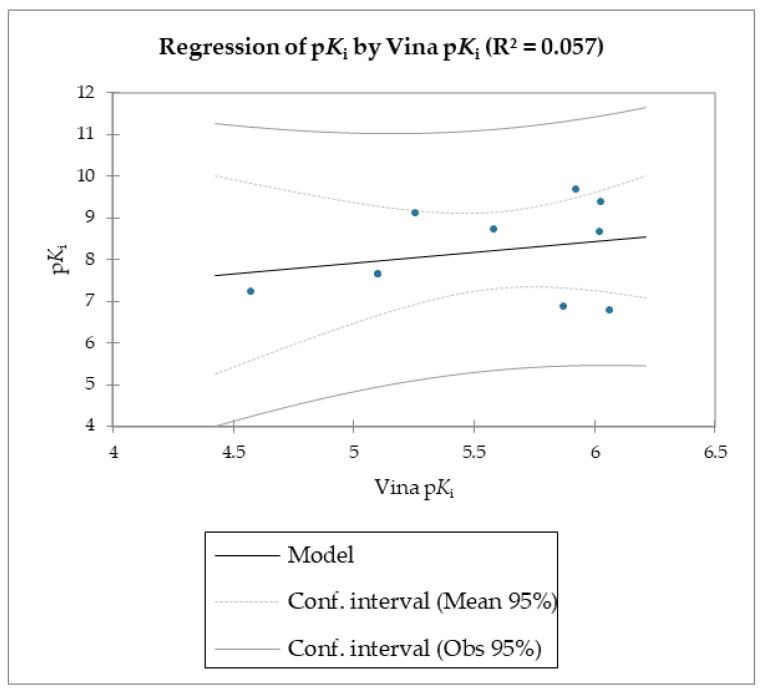
Regression of p*K*_i_ with Vina-calculated p*K*_i_.

The MMPB(GB)SA and the SMD simulations were superior overall at linearly correlating the experimental data (employing relative and not absolute binding value) for the studied molecules compared to the scoring functions of Autodock and Vina, which were the least able to correlate the calculated free energy with the experimental data (Table 2, Table 3 and Table 4, Figure 3 and Figure 4).

The reported data clearly shows the higher capability of SMD to score the affinities of small molecules against FABP4 compared to Autodock and Vina. In fact, as the value of the reference constant increased for each subset of molecules, we consistently obtained the increase of F_max_ required to extract the ligand from its receptor, following a linear correlation. Conversely, the Autodock and Vina scoring functions were not as accurate as the SMD data in potency evaluation, as they showed poor correlation between the increased potency of the compounds and the calculated binding energies.

To compare the SMD simulations’ energy evaluation with a more accurate energy of binding calculation and also include the effect of solvation, the experimental values were also compared to the MM-PB(GB)SA binding energies.

SMD simulation was recently shown to be as accurate as the MM/PBSA and molecular mechanics generalized Born surface area (MM/GBSA) techniques in predicting the binding affinity of small ligands to proteins. If a pulling speed of *v* = 0.005 nm/ps is used, then SMD simulation is computationally around 30-fold quicker than the MM/PBSA method, indicating its considerable potential for drug design [37]. Previous research found a strong link between F_max_ values and experimental free energies from 5 up to 20 protein–ligand systems, proving that a small dataset helps evaluate such a tool [37,44].

**Figure 5:**
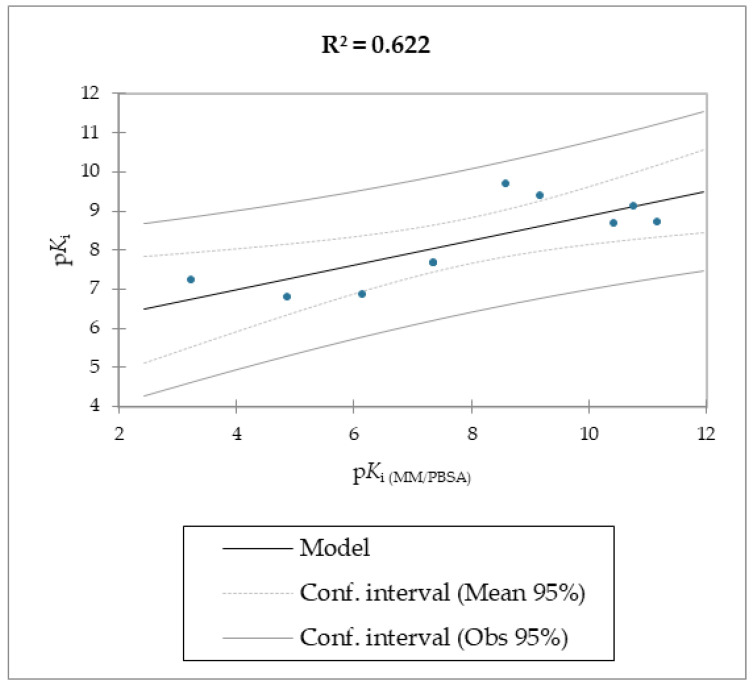
Calculated p*K*_i_ with MM/PBSA method vs. experimental p*K*_i_ of molecules **1**–**9**.

**Figure 6:**
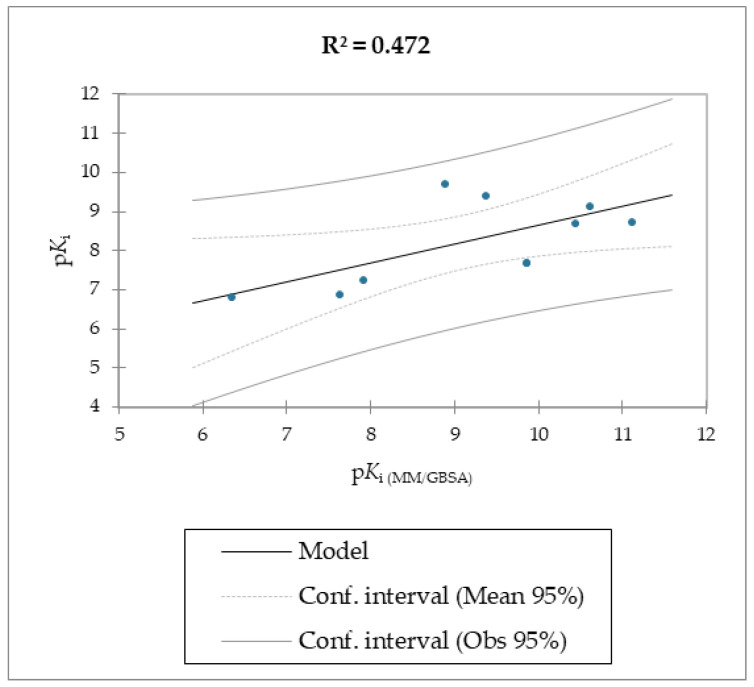
Calculated p*K*_i_ with MM/GBSA method vs. experimental p*K*_i_ of molecules **1**–**9**.

**Figure 7:**
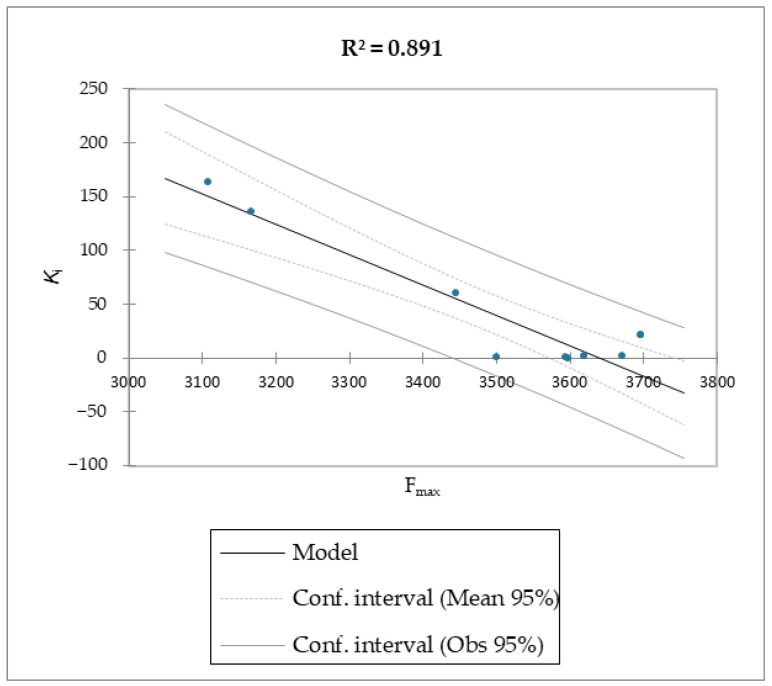
Calculated SMD simulation F_max_ vs. experimental *K*_i_ of molecules **1**–**9**.

The correlation between the experimental and calculated p*K*_i_ using the MM/PB(GB)SA and the SMD F_max_ methods are reported in Figure 5, Figure 6 and Figure 7.

Regarding molecules **1**–**9**, MM/PBSA and MM/GBSA’s scoring functions underperformed the SMD-derived data. Notably, the F_max_ overperformed all of the other data with a linear relationship and an R^2^ of 0.891, whereas the MM/PBSA and MM/GBSA models resulted in R^2^ values of 0.622 and 0.472, respectively. The three linear models were calculated using linear regression. The measured *K*_i_ was used as a dependent variable for the SMA-derived model, and the SMA F_max_ was used as the explanatory variable. Regarding the two models derived from the MM/PBSA and MM/GBSA models’ calculations, the experimental p*K*_i_ was used as a dependent variable, and the p*K*_i_, which was calculated using the MM/PBSA and MM/GBSA models, was used as the explanatory variable. The predictive capabilities for the last two models, measured as R^2^, were 0.622 and 0.472 for MM/PBSA and MM/GBSA, respectively, confirming the data model from the F_max_ linear model was better than the others derived from the MM/PBSA and MM/GBSA models’ calculations. The evaluation of the models was retrieved through several standard ways, as reported in Table 5.

The three equations calculated from the models are:

(1) $$pK_{i}=5.70572881585216+0.317475699514489\times pK_{i\;\left(\mathrm{MM}/\mathrm{PBSA}\right)}$$

(2) $$pK_{i}=3.79938746554068+0.485623403543389\times pK_{i\;\left(\mathrm{MM}/\mathrm{GBSA}\right)}$$

(3) $$K_{i}=1026.83687658054-0.281997931877324\times F_{max}$$

**Figure 8:**
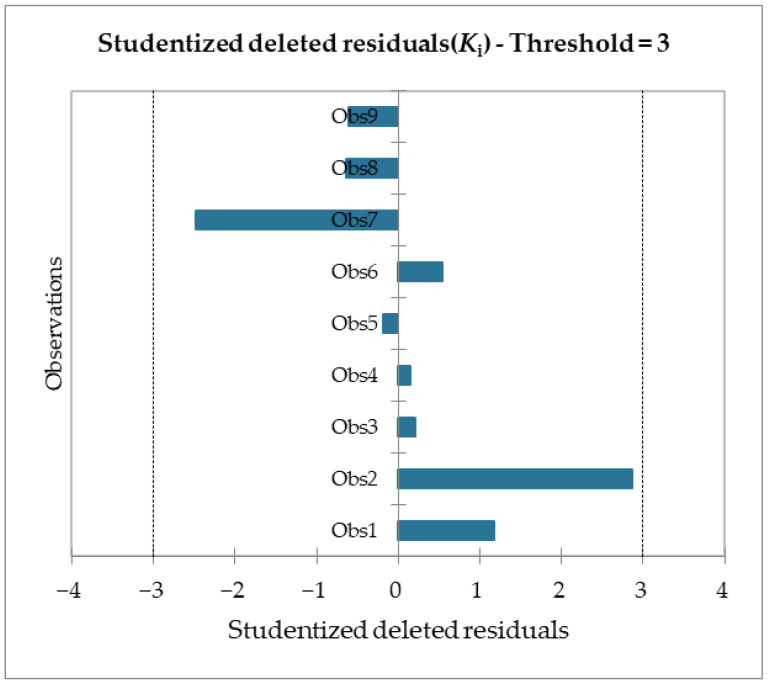
Studentized deleted residuals for the F_max_ model regression.

**Figure 9:**
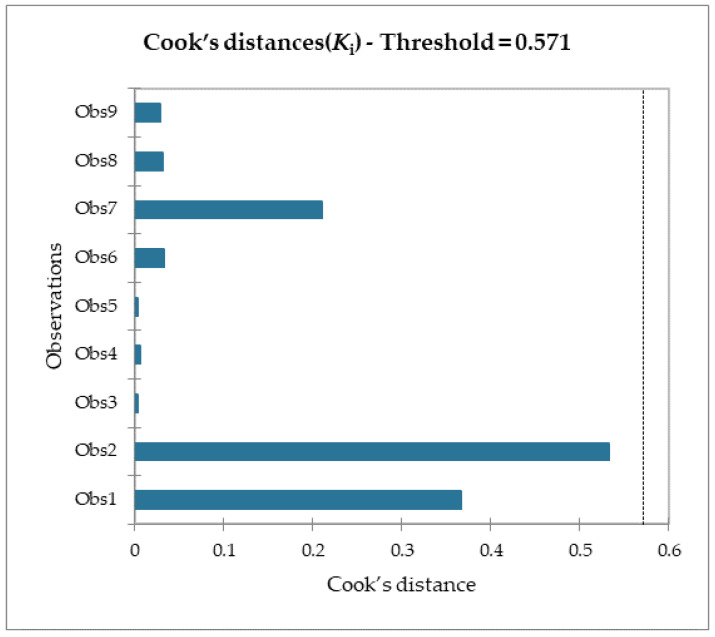
Cook’s distance for the F_max_ model regression.

Moreover, given the R^2^ of the F_max_ model, 89% of the variability of the dependent variable is explained by the explanatory variable. Considering the p-value of the computed F statistic shown in the ANOVA table (Table 6) and given a significance level of 5%, the information brought by the explanatory variables is significantly better than what a basic mean would bring. Outliers were checked with studentized deleted residuals and Cook’s distances, as reported in Figure 8 and Figure 9, and both confirmed the presence of no outliers among our training data.

Finally, to verify the predictive capabilities of the F_max_ model toward low-activity and inactive compounds, a second set of molecules was used; the structures of the molecules are reported in Table 7. Molecule **10** (used as a negative control with an experimental p*K*_i_ of 4.3) was outlined as the least active, with the lowest calculated SMD energy of 337.71 kcal/mol and an F_max_ of 2387.26 pN. Another five molecules were added to this second set as true negatives. Molecules **11**–**15** (decoy compounds) were generated by employing the DUD-E webserver and using BMS309403 (a FABP4i) as the reference compound [45]. The decoy compounds have similar physicochemical properties but different 2D topologies, i.e., they will be inactive in the same binding pocket. Interestingly, all of the molecules were identified as low-energy FABP4 binders in the SMD data, which was confirmed by the low F_max_ for all of the compounds, demonstrating the ability of the model to identify true negative compounds.

To exploit the new model in drug design, we generated a novel series of FABP4is and evaluated them with the proposed F_max_-based approach by using the computer-assisted scaffold hopping technique.

**Figure 10:**
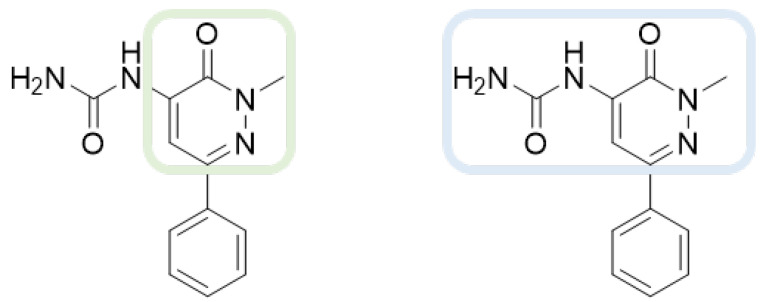
Scaffold hopping for the pyridazin-3-(2*H*)-one core.

**Figure 11:**
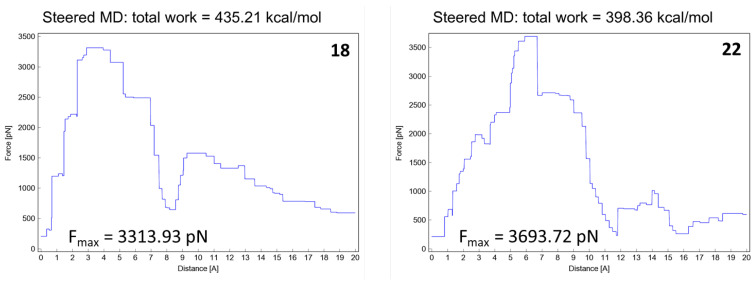
Total energies and F_max_ from SMD experiments (20 Å) for molecules **18** and **22**.

As shown in Figure 10, we focused on the search for bioisosteric-replacements/scaffold hopping in the central core of a recently published novel FABP4i scaffold with a pyridazin-3-(2*H*)-one central core [46]. Our bioisosteric replacement analysis led to 500 novel molecules for each series. Among the best compounds, as scored by the generated field similarity analysis (Tables S1 and S2), the 3-methoxy-6-phenylpyridazin-4-amine core was identified as a common one (compounds **18** and **22**) when considering the results of both series (Table 8). The newly identified core was then evaluated by using SMD-based calculations (Figure 11). Both compounds, **18** and **22**, were identified as promising FABP4is with calculated F_max_ values of 3313.93 and 3693.72 pN, respectively.

## 3. Materials and Methods

### 3.1. Ligand Selection

Different ligands were chosen for each set of structural affinities and tabulated according to the type of experimentally obtained equilibrium constants taken. A total of 14 molecules were selected and tested, each known in the literature to have complexed with the human FABP4 protein [22]. Pairwise similarity was calculated by using circular fingerprints and by using Flare v 6.1 (Cresset Biomolecular Discovery Ltd., Cambridge, Cambridgeshire, UK) [43]. Tanimoto similarity was calculated with ChemMine Tools (https://chemminetools.ucr.edu/, accessed on 14 March 2023).

### 3.2. Initial Configuration

All of the ligand structures were initially minimized using Marvin Sketch (18.24, ChemAxon Ltd., Budapest, Hungary) to obtain a system configured for SMD simulation. All of the structures were subjected to molecular mechanics energy minimization using the MMFF94 force field [47]. The 3D geometry of all compounds was then optimized using the PM3 Hamiltonian method [48] as implemented in the MOPAC 2016 package (MOPAC2016 v. 18.151, Stewart Computational Chemistry, Colorado Springs, CO, USA) and assuming a pH of 7.0 [49]. Each structure was then docked to the human FABP4 protein PDB code, 6LJX, with a resolution of 1.75 Å. At the end of the docking study, the most stable pose presenting the receptor-ligand interaction, as shown by the PDB model, was chosen. Only one FABP4 protein structure over the several structures present in the PDB was chosen after evaluating the possible differences between the structures. Twenty different proteins (Figure S2) were selected, and the RMSD was calculated after the alignment of the 3D structures. An overall RMSD of 0.274 Å was calculated, demonstrating that the proteins were almost identical and that the different ligands in the binding pocket did not influence the 3D structures. Moreover, we performed 100 ns of MD simulation on three of them to further investigate the variation of the structures over time. An overall RMSD of 1.154 Å was calculated during the simulation while considering the average structures, minimum energy structures, and structures at the end of the 100 ns of MD simulation (Figure S3).

Flexible ligand docking experiments were performed using the Autodock (4.2.6) or Vina (1.1.2) software implemented in YASARA (v. 22.9.24, YASARA Biosciences GmbH, Vienna, Austria) and using the three-dimensional crystal structures of the human FABP4 (PDB ID: 6LJX) obtained from the Protein Data Bank and the Lamarckian Genetic Algorithm (LGA) as previously described [50,51,52,53]. The protein was protonated and optimized with YASARA. Maps were generated by using AutoGrid (4.2.6) with a spacing of 0.375 Å and sizes that included all atoms extending 7 Å from the surface of the Arg147 amino acid of the crystallized ligand. The point charges were initially assigned based on the AMBER03 force field and then damped to mimic the less polar Gasteiger charges used to optimize Autodock’s scoring function. All parameters were entered with default settings, as previously reported. In the docking tab, the macromolecule and ligand were selected, and the LGA parameters were set as follows: ga_runs = 100, ga_pop_size = 150, ga_num_evals = 25,000,000, ga_num_generations = 27,000, ga_elitism = 1, ga_mutation_rate = 0. 02, ga_crossover_rate = 0.000, ga_crossover_rate = 0.000. 02, ga_crossover_rate = 0.8, ga_crossover_mode = two points, ga_cauchy_alpha = 0.0, ga_cauchy_beta = 1.0, and number of generations for selection of the worst individual = 10. The capability of each docking protocol to obtain a reliable binding pose for each model was validated by comparing the best-docked pose and the real crystallized poses inside FABP4 for the following molecules, which were retrieved from the protein data bank: 2NNQ, 5HZ6, 5EDC, 5Y12, 5Y0X, 6LJV, 6LJU, and 6LJS. All of the calculated binding poses were compared with those retrieved from the protein data bank, resulting in a calculated RMSD < 1.00 Å.

MM/PB(GB)SA rescoring procedures were obtained by using fastDRH as an open-access web server (http://cadd.zju.edu.cn/fastdrh/overview, accessed on 5 December 2022) [54].

### 3.3. Steered Molecular Dynamics, Data Analysis, and Isosteric Replacement

For each protein-ligand system, a pull vector was defined, with the initial coordinates being the center of mass of the ligand. The reaction coordinate was defined as the projection of the distance vector between the Cartesian coordinates of the center of mass of the ligand to the pull vector. The setup included optimizing the hydrogen bond network to increase the stability of the solute and predicting the p*K*_a_ to fine-tune the protonation states of the protein residues at the chosen pH of 7.4, as in the SMD macro in YASARA [55,56]. NaCl ions were added at a physiological concentration of 0.9 percent, with an excess of Na or Cl used to neutralize the cell. After the steepest descent and simulated annealing minimizations to remove the clashes, the simulation was run for the picoseconds required for the 20 Å distance to be traveled (50–150 ps) by using the AMBER14 force field for the solute, GAFF2 and AM1BCC for the ligands, and TIP3P for the water. The cutoff was 8 Å for Van der Waals forces, whereas no cutoff was applied to electrostatic forces. The equations of motion were integrated with multiple timesteps of 1.25 fs for bound interactions and 2.5 fs for unbound interactions at a temperature of 298 K and a pressure of 1 atm. After an equilibration time of 3 ps, the SMD perturbation started with a minimum acceleration of 2000 pm/ps^2^ applied to all ligand atoms and unbound forces (every 2.5 fs). Considering the mass of the ligand to be (X) Daltons and the equation F = *m* × *a*, a tensile force of (2000 × X × 0.00166) pN was obtained. The pulling direction was considered the vector connecting the receptors’ centers of mass. It was provided manually to drag all ligands uniformly from the interaction pocket, and it was continuously updated to account for the rotations of the complex. The maximum distance between the receptor and ligand’s centers of mass was continuously updated, and if it did not increase by 400 steps, then acceleration was increased by 500 pm/ps^2^. As soon as the maximum distance increased with a MaxDisSpeed above 4000 m/s, i.e., a barrier was crossed, and nothing prevented the ligand from accelerating, the acceleration was reduced by a factor of 1−(1–4000/MaxDisSpeed)^2^ but not below the initial minimum. This check was performed every 20 simulation steps. The simulation was stopped when the ligand traveled 20 Å from the starting position and ultimately exited the receptor pocket. The peak pulling force and total work done were calculated to correlate with the binding force visible in the presented graphs. All simulations were performed using the YASARA (v. 22.9.24, YASARA Biosciences GmbH, Vienna, Austria) software simulation package. XLSTAT (v. 2021.4.1 by Addinsoft, Paris, Ile-de-France, France) was used for regression analysis. The structures for the isosteric replacement were built as already described for the others. Once built and optimized, all structures were used in the bioisostere replacement tool Spark 10.4.0 (Cresset Biomolecular Discovery Ltd., Cambridge, Cambridgeshire, UK). Five hundred compounds were generated for the substitution (the fifty best compounds reported in the Supplementary Materials). The isosteric replacement was performed using the same 178,558 fragments for each part; notably, the fragments derived from ChEMBL and Zinc databases had a protocol that was already reported and validated [27,57].

The run using umbrella sampling (US) methodology on molecule **6** was carried out employing the YASARA macro written by Silva (https://www.researchgate.net/post/Do_you_need_scripts_for_umbrella_sampling_simulations_for_use_with_YASARA, accessed on 28 February 2023) [58].

## 4. Conclusions

Inhibiting FABP4 is a viable and appealing therapeutic opportunity for treating metabolic disorders [59,60,61,62]. Furthermore, given the discovery of the protein’s role in cancer progression, the inhibition of FABP4 might offer a viable therapeutic option for cancer patients through the suppression or decrease of early-stage tumors and metastasis, and they have a possible use as biomarkers for cancer detection [63,64,65,66,67,68]. Nevertheless, no FABP4i has entered the clinical research phase so far. This is mainly due to several unavoidable adverse effects of FAPBis, including metabolic issues, in vivo toxicity, and rapidly acquired drug resistance [69]. Believing that this family of transporter proteins holds promise as a valid therapeutic target, research must still try to pursue the common aim of bringing FAPB inhibitors into clinics.

Computational approaches were used to identify novel scaffolds for FABP4 inhibition. Our research group stayed widely active in this field by applying several structures and ligand-based computational tools in FABP4is research [14,22,25,26,27,46]. SMD simulation was never employed to study and rank small-ligand FABP4is.

SMD simulations can be used to evaluate the conformation, stability, and interactions of proteins with surrounding macromolecules (membrane, DNA, RNA, or other proteins). Moreover, SMD is a solid and practical approach for gaining insight into binding mechanisms and acquiring the relative binding energies between candidate drugs and targeted proteins by simply considering the mechanical components, such as ligands and target flexibility. In contrast to the majority of previously reported computational approaches, which focus on accurate binding energy calculations using MD simulations, SMD simulation has the potential to provide more effective ranking procedures while reducing computational expense when combined with existing methods for endpoint free energy calculations, such as molecular mechanics/Poisson–Boltzmann surface area, free energy perturbation, and thermodynamic integration. Thus, we reasoned that SMD simulation might play an essential role in FABP4is’ rational design.

For the first time, we established good agreement between SMD simulation-retrieved data and experimental inhibition or dissociation constants for a dataset of compounds targeting the FABP4 protein. Our study supports SMD simulation as a promising method for evaluating new small-molecule FABP4is. The key benefit of SMD simulation is that it is computationally less expensive than the MM/PB(GB)SA method and, in general, substantially more accurate than the docking approach and scoring functions. Moreover, this work proves SMD simulation is a valuable strategy for ranking novel FABP4is. It will consequently enlarge the arsenal of tools to assist medicinal chemists working in the field, as it demonstrates a practical approach for the future identification of FABP4is for clinical use.

In comparison to the MM/PB(GB)SA method, despite being highly accurate, one limit of the SMD-derived ranking is that it cannot directly predict absolute binding affinities. Another limitation is that other MD simulation-based experiments, such as the US method, could be more capable of calculating a ligand’s free energy of binding. US-method calculations were recently reported to be able to reliably estimate binding free energy for a complex of small molecules/proteins. Compared with SMD, the better results obtained with the US method come at the cost of computational resources. The total MD simulation time with the US method was recently reported to be as long as 120 ns of simulation [70], which is remarkably high compared to the 50–150 ps required with our SMD methodology (16 h vs. 9–12 min with an AMD Ryzen Threadripper PRO 5975WX equipped with a GeForce RTX 3060 Ti graphics card).

Instead, the computational method presented here can be regarded as a tool to help medicinal chemists pursue molecular modifications and new synthetic directions in FABP4is research that would have been too risky with no computational validation support. It can also be used to readily identify compounds that would be unlikely to meet the desired affinity [71]. This significantly lowers the risk of embarking on laborious synthetic protocols by anticipating if such a compound is/is not projected to accomplish the potency objectives (as informed by SMD ranking assessments), allowing researchers to focus on target molecules endowed with more promising characteristics as active FABP4is. Moreover, the SMD methodology was used to rank a newly designed library of FABP4is that was built from a biologically active heterocyclic framework recently identified through scaffold hopping replacement [46]. Synthetic procedures are being investigated to produce compounds **18** and **22** (with the 3-methoxy-6-phenylpyridazin-4-amine core) identified in this work, resulting in potentially valid FABP4is; this is definitely worth further biological study.

## Figures and Tables

**Table 1:** Structures of molecules **1**–**9**, experimental and calculated (Autodock, Vina, MM/PBSA, and MM/GBSA) p*K*_i_, total SMD energy, and SMD F_max_.

N	Structure	Exp. p*K*_i_	Autodock p*K*_i_	Vina p*K*_i_	MM/PBSA p*K*_i_	MM/GBSA p*K*_i_	Total SMD Energy (kcal/mol)	SMD F_max_ (pN)
**1**	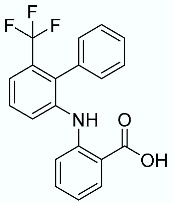	6.79	6.13	6.07	4.87	6.35	607.57	3107.35
**2**	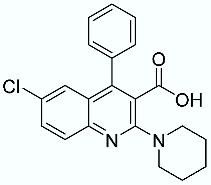	7.66	6.29	5.10	7.36	9.87	743.43	3696.76
**3**	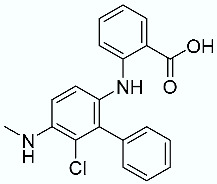	7.22	5.07	4.58	3.23	7.92	579.64	3444.44
**4**	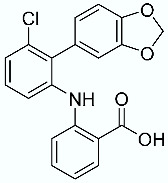	6.87	6.15	5.87	6.15	7.63	575.78	3167.07
**5**	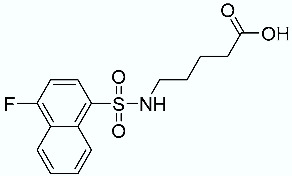	8.67	5.45	6.02	10.43	10.45	353.09	3619.763
**6**	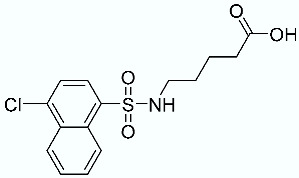	8.73	4.94	5.58	11.16	11.11	490.62	3671.292
**7**	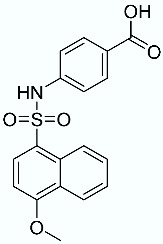	9.12	4.82	5.26	10.75	10.62	477.53	3500.413
**8**	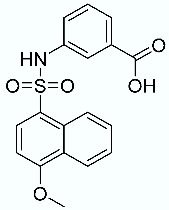	9.40	4.85	6.03	9.18	9.38	585.43	3594.054
**9**	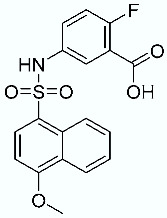	9.68	5.51	5.93	8.60	8.89	503.11	3596.79

**Table 2:** FABP4 experimental *K*_i_ and free energy of binding; Δ*G*_bind_ calculated with Autodock, Vina, MM/PBSA, and MM/GBSA (kcal/mol).

N	*K*_i_ (nM)	Calculated Δ*G*_bind_ from *K*_i_	Autodock Δ*G*_bind_	Vina Δ*G*_bind_	MM/PBSA Δ*G*_bind_	MM/GBSA Δ*G*_bind_
**1**	160.1	−9.26	−8.36	−8.27	−6.63	−8.66
**2**	22	−10.44	−8.57	−6.96	−10.04	−13.45
**3**	60	−9.84	−6.91	−6.24	−4.40	−10.79
**4**	136	−9.36	−8.38	−8.01	−8.39	−10.40
**5**	2.16	−11.81	−7.43	−8.21	−14.21	−14.25
**6**	1.85	−11.90	−6.74	−7.61	−15.22	−15.15
**7**	0.76	−12.43	−6.57	−7.17	−14.66	−14.47
**8**	0.40	−12.81	−6.62	−8.22	−12.51	−12.79
**9**	0.21	−13.19	−7.52	−8.08	−11.72	−12.12

**Table 3:** Goodness of fit statistics (Autodock p*K*_i_).

R²	0.424
Adjusted R²	0.342
MSE	0.822
RMSE	0.906
MAPE	7.863
DW	1.451
Cp	2.000
AIC	−0.029
SBC	0.365
PC	0.905
Press	8.411
Q²	0.158

**Table 4:** Goodness of fit statistics (Autodock p*K*_i_).

R²	0.057
Adjusted R²	−0.078
MSE	1.345
RMSE	1.160
MAPE	11.219
DW	0.682
Cp	2.000
AIC	4.405
SBC	4.800
PC	1.482
Press	15.000
Q²	−0.502

**Table 5:** Goodness of fit statistic for MM/PBSA, MM/GBSA, and F_max_ models.

	MM/PBSA	MM/GBSA	F_max_
R²	0.622	0.472	0.891
Adjusted R²	0.568	0.396	0.875
MSE	0.539	0.753	511.691
RMSE	0.734	0.868	22.621
MAPE	6.814	7.281	1684.485
DW	0.989	0.509	1.406
AIC	2.000	2.000	57.878
SBC	−3.819	−0.810	58.272
PC	−3.425	−0.415	0.172
Press	0.594	0.830	5709.609
Q²	5.871	7.204	0.826

**Table 6:** Variance table of F_max_ model. Pr: *p*-value for F statistics.

Source	DF	Sum of Squares	Mean Squares	F	Pr > F
Model	1	29,229.336	29,229.336	57.123	0.00013
Error	7	3581.838	511.691		
Corrected Total	8	32,811.174			

**Table 7:** Molecules **10** (true negative) and **11**–**15** (decoy compounds) and their SMD-derived data.

N	Structure	Total SMD Energy (kcal/mol)	SMD F_max_ (pN)
**10**	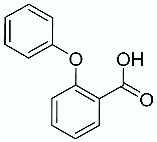	337.71	2387.26
**11**	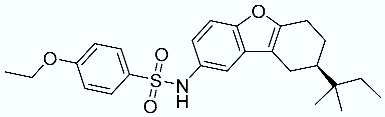	285.87	1523.115
**12**	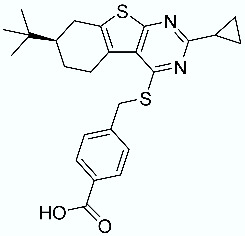	324.15	2178.367
**13**	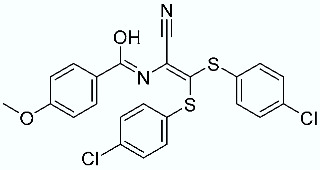	349.20	1707.508
**14**	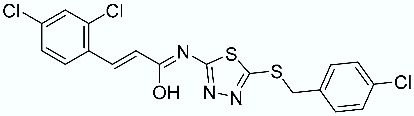	261.82	1494.272
**15**	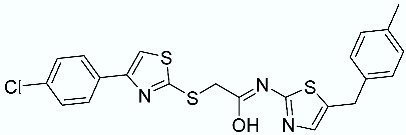	251.42	1565.923

**Table 8:** Best five compounds from the scaffold hopping replacement.

**N**	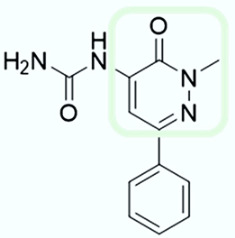	**N**	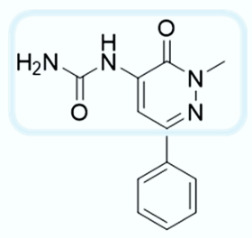
**16**	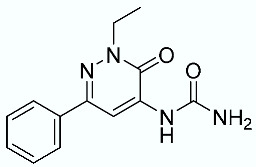	**21**	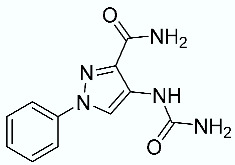
**17**	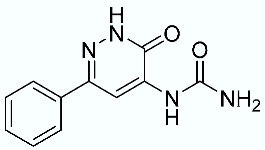	**22**	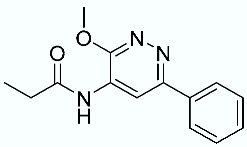
**18**	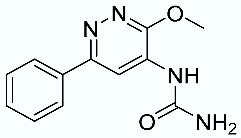	**23**	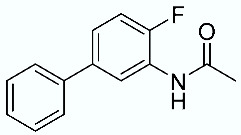
**19**	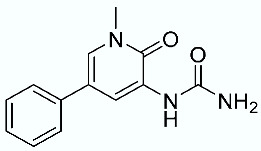	**24**	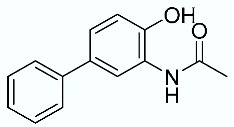
**20**	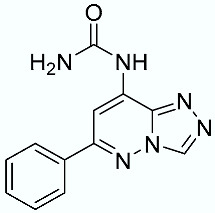	**25**	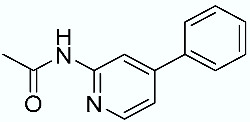

## Data Availability

Not applicable.

## Supplementary Materials

The following supporting information can be downloaded at: https://www.mdpi.com/article/10.3390/molecules28062731/s1. Figure S1: Similarity matrix calculated by Tanimoto index for molecules **1**–**9**; Figure S2: RMSD matrix for the selected FABP4 from the Protein Data Bank; Figure S3: RMSD matrix for selected structures during 100 ns of MD; Table S1: Molecules from the first scaffold hopping replacement; Table S2: Molecules from the second scaffold hopping replacement.
