# Supplementary material for: Steered Molecular Dynamics Simulations Study on FABP4 Inhibitors
Source: Molecules. 2023 Mar 17;28(6):2731. doi: 10.3390/molecules28062731 (PMC10058326; doi:10.3390/molecules28062731)
Supplement: Supplementary file 1 [file molecules-28-02731-s001.zip › molecules-2229034-supplementary.pdf]

molecules

MDPI

Supplementary Material

Steered molecular dynamics simulations study on FABP4 inhibitors

Rosario Tomarchio [1,†], Vincenzo Patamia [1,†], Chiara Zagni [1], Letizia Crocetti [2], Agostino Cilibrizzi [3,4], Giuseppe Floresta [1,*] and Antonio Rescifina [1,*]

[1] Department of Drug and Health Sciences, University of Catania, Viale A. Doria 6, 95125 Catania, Italy; vincenzo.patamia@unict.it (V.P.) rosario.tomarchioct@gmail.it (R.T.), chiara.zagni@unict.it (C.Z.), giuseppe.floresta@unict.it (G.F.), arescifina@unict.it (A.R.).

[2] Department NEUROFARBA – Pharmaceutical and Nutraceutical Section, via Ugo Schiff 6, 50019 Sesto Fiorentino (FI), Italia; letizia.crocetti@unifi.it (L.C.).

[3] Institute of Pharmaceutical Science, King's College London, Stamford Street, London, SE1 9NH, UK; agostino.cilibrizzi@kcl.ac.uk (A.C.)

[4] Centre for Therapeutic Innovation, University of Bath, Bath, UK

† These authors contributed equally

* Correspondence: giuseppe.floresta@unict.it (G.F.) arescifina@unict.it (A.R.)

Table of content

Figure S1. Similarity matrix calculated by Tanimoto index for molecules **1–9** S2
Figure S2. RMSD matrix for the selected FABP4 from the Protein Data Bank S3
Figure S3. RMSD matrix for selected structures during 100 ns of MD S4
Table S1. Molecules from the first scaffold hopping replacement S4
Table S2. Molecules from the second scaffold hopping replacement S5

Tanimoto

	1	2	3	4	5	6	7	8	9
1		0.300	0.759	0.733	0.143	0.143	0.214	0.214	0.209
2	0.300		0.342	0.268	0.231	0.263	0.214	0.214	0.209
3	0.759	0.342		0.821	0.146	0.175	0.220	0.220	0.214
4	0.733	0.268	0.821		0.143	0.171	0.214	0.214	0.209
5	0.143	0.231	0.146	0.143		0.913	0.469	0.469	0.455
6	0.143	0.263	0.175	0.171	0.913		0.469	0.469	0.455
7	0.214	0.214	0.220	0.214	0.469	0.469		0.786	0.759
8	0.214	0.214	0.220	0.214	0.469	0.469	0.786		0.962
9	0.209	0.209	0.214	0.209	0.455	0.455	0.759	0.962	

High similarity

Low similarity

Figure S1. Similarity matrix calculated by Tanimoto index for molecules **1–9**.

	1	2	3	4	5	6	7	8	9	10	11	12	13	14	15	16	17	18	19	20
1: 5D48.A	0.00	0.35	0.34	0.29	0.27	0.28	0.27	0.28	0.29	0.34	0.28	0.26	0.24	0.25	0.25	0.25	0.28	0.19	0.46	0.23
2: 5Y0F.A	0.35	0.00	0.05	0.20	0.24	0.18	0.19	0.21	0.21	0.38	0.33	0.27	0.29	0.32	0.31	0.28	0.33	0.29	0.50	0.30
3: 5Y0G.A	0.34	0.05	0.00	0.19	0.24	0.17	0.18	0.21	0.21	0.38	0.31	0.26	0.29	0.32	0.31	0.27	0.32	0.28	0.50	0.29
4: 6LJS.A	0.29	0.20	0.19	0.00	0.12	0.09	0.09	0.10	0.14	0.33	0.27	0.21	0.23	0.23	0.21	0.25	0.26	0.21	0.45	0.24
5: 6LJT.A	0.27	0.24	0.24	0.12	0.00	0.11	0.11	0.13	0.19	0.31	0.29	0.23	0.23	0.21	0.21	0.25	0.23	0.19	0.42	0.24
6: 6LJU.A	0.28	0.18	0.17	0.09	0.11	0.00	0.09	0.11	0.15	0.33	0.26	0.20	0.22	0.24	0.23	0.23	0.25	0.20	0.45	0.23
7: 6LJV.A	0.27	0.19	0.18	0.09	0.11	0.09	0.00	0.11	0.15	0.31	0.29	0.22	0.22	0.20	0.19	0.23	0.24	0.19	0.42	0.22
8: 6LJW.A	0.28	0.21	0.21	0.10	0.13	0.11	0.11	0.00	0.17	0.35	0.26	0.22	0.22	0.23	0.22	0.24	0.26	0.20	0.45	0.25
9: 6LJX.A	0.29	0.21	0.21	0.14	0.19	0.15	0.15	0.17	0.00	0.32	0.26	0.20	0.22	0.25	0.22	0.22	0.26	0.21	0.45	0.22
10: 2NNQ.A	0.34	0.38	0.38	0.33	0.31	0.33	0.31	0.35	0.32	0.00	0.41	0.36	0.39	0.33	0.32	0.35	0.34	0.32	0.41	0.34
11: 3P6D.A	0.28	0.33	0.31	0.27	0.29	0.26	0.29	0.26	0.26	0.41	0.00	0.16	0.19	0.34	0.32	0.23	0.32	0.24	0.52	0.20
12: 3P6F.A	0.26	0.27	0.26	0.21	0.23	0.20	0.22	0.22	0.20	0.36	0.16	0.00	0.15	0.28	0.27	0.18	0.27	0.20	0.48	0.19
13: 3P6H.A	0.24	0.29	0.29	0.23	0.23	0.22	0.22	0.22	0.22	0.39	0.19	0.15	0.00	0.25	0.24	0.21	0.25	0.17	0.46	0.17
14: 4NNS.A	0.25	0.32	0.32	0.23	0.21	0.24	0.20	0.23	0.25	0.33	0.34	0.28	0.25	0.00	0.09	0.26	0.25	0.19	0.35	0.24
15: 4NNT.A	0.25	0.31	0.31	0.21	0.21	0.23	0.19	0.22	0.22	0.32	0.32	0.27	0.24	0.09	0.00	0.25	0.25	0.19	0.36	0.23
16: 5D4A.A	0.25	0.28	0.27	0.25	0.25	0.23	0.23	0.24	0.22	0.35	0.23	0.18	0.21	0.26	0.25	0.00	0.24	0.17	0.46	0.17
17: 5D45.A	0.28	0.33	0.32	0.26	0.23	0.25	0.24	0.26	0.26	0.34	0.32	0.27	0.25	0.25	0.25	0.24	0.00	0.17	0.46	0.25
18: 5D47.A	0.19	0.29	0.28	0.21	0.19	0.20	0.19	0.20	0.21	0.32	0.24	0.20	0.17	0.19	0.19	0.17	0.17	0.00	0.42	0.16
19: 5HZ6.A	0.46	0.50	0.50	0.45	0.42	0.45	0.42	0.45	0.45	0.41	0.52	0.48	0.46	0.35	0.36	0.46	0.46	0.42	0.00	0.43
20: 3RZY.A	0.23	0.30	0.29	0.24	0.24	0.23	0.22	0.25	0.22	0.34	0.20	0.19	0.17	0.24	0.23	0.17	0.25	0.16	0.43	0.00

4.0
3.5
3.0
2.5
2.0
1.5
1.0
0.5
0.0

Figure S2. RMSD matrix for the selected FABP4 from the Protein Data Bank (PDBids are reported in the Figure).

	1	2	3	4	5	6	7	8	9
1: 6LJX ligand in 6LJX protein: average	0.00	0.61	0.64	0.84	0.55	1.93	0.24	0.68	0.76
2: 6LJX ligand in 6LJX protein: energy min	0.61	0.00	0.87	1.14	0.48	2.05	0.66	0.50	0.90
3: 6LJX ligand in 6LJX protein: 100 ns	0.64	0.87	0.00	0.95	0.74	1.83	0.75	0.89	1.12
4: 6LJX ligand in 2NNQ protein: average	0.84	1.14	0.95	0.00	1.02	1.35	0.91	1.09	1.19
5: 6LJX ligand in 2NNQ protein: energy min	0.55	0.48	0.74	1.02	0.00	1.94	0.63	0.51	0.95
6: 6LJX ligand in 2NNQ protein: 100 ns	1.93	2.05	1.83	1.35	1.94	0.00	1.98	1.99	2.10
7: 6LJX ligand in 6LJV protein: average	0.24	0.66	0.75	0.91	0.63	1.98	0.00	0.73	0.64
8: 6LJX ligand in 6LJV protein: energy min	0.68	0.50	0.89	1.09	0.51	1.99	0.73	0.00	1.00
9: 6LJX ligand in 6LJV protein: 100 ns	0.76	0.90	1.12	1.19	0.95	2.10	0.64	1.00	0.00

Scale: 4.0, 3.5, 3.0, 2.5, 2.0, 1.5, 1.0, 0.5, 0.0

Figure S3. RMSD matrix for the selected FABP4 structures from the PDB (PDBids are reported in the Figure). Average structure during MD simulation, minimum energy structure during the simulation, and final structure at 100 ns of simulation.

Table S1. Molecules from the first scaffold hopping replacement.

Rank	Structure	Field Score
molecule (**16**) 1	O=C1C(NC(=O)N)=CC(=NN1CC)c2ccccc2	0.963
molecule (**17**) 2	O=C1C(NC(=O)N)=CC(=NN1)c2ccccc2	0.935
molecule (**18**) 3	O=C(Nc1cc(nnc1OC)-c2ccccc2)N	0.911
molecule (**19**) 4	O=C(NC1=CC(=CN(C1=O)C)c2ccccc2)N	0.873
molecule (**20**) 5	O=C(Nc1cc(nn2cnnc12)-c3ccccc3)N	0.882
6	O=C(N)/C=C/1C=C(C=C(C1=O)C)c2ccccc2	0.853
7	O=C(Nc1cc(nn2c(nnc12)C)-c3ccccc3)N	0.894
8	O=C(Nc1cc(nc(OC)n1)-c2ccccc2)N	0.883
9	O=C(N)Cc1cc(nc(n1)C)-c2ccccc2	0.863
10	O=C(Nc1cc(nc(n1)C)-c2ccccc2)N	0.859
11	O=C(Nc1cc(nc([n+]1[O-])N)-c2ccccc2)N	0.829
12	O=C(Nc1cc(nc2ccnn21)-c3ccccc3)N	0.859
13	Clc1nc(NC(=O)N)cc(n1)-c2ccccc2	0.856
14	O=C(Nc1cc(nc(n1)CC)-c2ccccc2)N	0.857
15	Oc1nc(NC(=O)N)cc(n1)-c2ccccc2	0.837
16	O=C1C(NC(=O)N)=C(C(=NN1C)c2ccccc2)C=C	0.879
17	O=C(Nc1cc(nc(SC)n1)-c2ccccc2)N	0.856
18	O=C(n1c2cc(nc(N)c2nn1)-c3ccccc3)N	0.827
19	O=C1/C(C(=NN1)c2ccccc2)=C\NC(=O)N	0.855
20	O=C(Nc1cc(nc(N)c1[N+]([O-])=O)-c2ccccc2)N	0.846
21	O=C(Nc1cc(n2c(n1)ccn2)-c3ccccc3)N	0.85
22	O=C(N)Cc1cc(ncn1)-c2ccccc2	0.85
23	O=C1C(NC(=O)N)=CC(c2ccccc2)=CN1	0.839

24	O=C(Nc1cc(ncn1)-c2ccccc2)N	0.847
25	O=C(Nc1cc(nc(n1)NC)-c2ccccc2)N	0.842
26	O=C(Nc1cc(nc(n1)N)-c2ccccc2)N	0.82
27	O=C(Oc1cc(nc(n1)C)-c2ccccc2)N	0.823
28	O=C(Nc1cc(nc(c1C#N)C)-c2ccccc2)N	0.825
29	O=C(c1cc(nn1CC)-c2ccccc2)C(=O)N	0.839
30	O=C(Nc1cc(nc(c1)C)-c2ccccc2)N	0.81
31	O=C(N)Cc1cc(nc(O)n1)-c2ccccc2	0.81
32	O=C(Nc1cc(nc2cc(nn21)C)-c3ccccc3)N	0.851
33	O=C(Nc1cn(nc1CC)-c2ccccc2)N	0.831
34	O=C1C(c2ccccc2)=CC(OC(=O)N)=NN1C	0.816
35	O=C(Nc1cc(nc(n1)C2CC2)-c3ccccc3)N	0.864
36	O=C1C(NC(=O)N)=CC(c2ccccc2)=C(O1)C	0.845
37	Clc1nc(OC(=O)N)cc(n1)-c2ccccc2	0.814
38	O=C(/C=C(/OC(=O)N)C)c1ccccc1	0.847
39	O=C(Nc1cc(cc(n1)C)-c2ccccc2)N	0.789
40	FC(F)(F)c1nc(NC(=O)N)cc(n1)-c2ccccc2	0.844
41	O=C(Nc1cc(nc2c1cc[nH]2)-c3ccccc3)N	0.812
42	O=C(Nc1cc(nc(n1)N(C)C)-c2ccccc2)N	0.842
43	O=C(Nc1cc(nc(n1)COC)-c2ccccc2)N	0.839
44	Clc1cc(NC(=O)N)cc(n1)-c2ccccc2	0.799
45	O=C(N)/C=C(/C(OC)=O)C(Oc1ccccc1)=O	0.817
46	O=C(N)c1cnc2c(c(nn2C)-c3ccccc3)c1	0.813
47	O=C(Nc1cc(nc(n1)C(C)C)-c2ccccc2)N	0.853
48	O=C(Nc1c(C)cnc(-c2ccccc2)c1)N	0.812
49	O=C(N)/C=C\1C(OC(c2ccccc2)=C1)=O	0.836
50	O=C(NC=1C=C(C(=O)N(C1)C)c2ccccc2)N	0.791

Table S2. Molecules from the second scaffold hopping replacement.

Rank	Structure	Field Score
molecule (**21**) 1	O=C(N)c1c(NC(=O)N)cn(n1)-c2ccccc2	0.803
molecule (**22**) 2	O=C(Nc1cc(nnc1OC)-c2ccccc2)CC	0.786
molecule (**23**) 3	Fc1ccc(cc1NC(=O)C)-c2ccccc2	0.756
molecule (**24**) 4	Oc1ccc(cc1NC(=O)C)-c2ccccc2	0.748
molecule (**25**) 5	O=C(Nc1cc(-c2ccccc2)ccn1)C	0.77
6	Clc1ccc(cc1NC(=O)C)-c2ccccc2	0.761
7	CO/N=C(/c1ccccc1)c2ccon2	0.818
8	O=C(Nc1cccc(-c2ccccc2)c1)N	0.745
9	O=C(Nc1cccc(-c2ccccc2)c1)C	0.733
10	Oc1ccc(-c2ccccc2)cc1NC=O	0.747
11	O=CNc1cccc(-c2ccccc2)c1	0.749
12	O=C(Nc1cc(-c2ccccc2)ccc1OC)C	0.739
13	O=C(N)c1cc(nc2ccccc21)-c3ccccc3	0.762
14	n12cnnc1c(nc3ccccc32)-c4ccccc4	0.791
15	O=C(Nc1cc(-c2ccccc2)ccn1)CC	0.755
16	O=C1c2ccccc2C(=NN1C)c3ccccc3	0.732
17	O=C(Nc1cncc(c1)-c2ccccc2)C	0.722

18	Nc1cc2c(c(ncn2)-c3ccccc3)cn1	0.751
19	O=C(N)c1cc2c(s1)c(ncn2)-c3ccccc3	0.781
20	O=C(Nc1cccc(-c2ccccc2)c1)CC	0.744
21	O=C(Nc1cc(-c2ccccc2)ccn1)C3CC3	0.766
22	O=C1NN=C(N1/N=C/c2ccccc2)C	0.782
23	O=C1C=2CCCCC2C(=NN1C)c3ccccc3	0.713
24	Oc1ccc(c2c1NC(S2)=O)-c3ccccc3	0.745
25	Clc1nc(nc(NCC)n1)-c2ccccc2	0.706
26	Cc1nc(-c2ccccc2)cc(-n3cccn3)n1	0.726
27	Cc1nc(cc(NC2CC2)n1)-c3ccccc3	0.691
28	CN(c1nc(OC)nc(n1)-c2ccccc2)C	0.716
29	Oc1ccc(NC(=O)C)cc1-c2ccccc2	0.718
30	O=C(c1ccccc1)c2nnc(s2)N	0.789
31	Clc1cc(cc(CC([O-])=O)c1)-c2ccccc2	0.706
32	Oc1c(cc(cc1C)-c2ccccc2)C	0.723
33	O=C1C(N)=C(C(=NN1c2ccccc2)C)C=C	0.764
34	O=C1c2cc(ncc2N=CN1)-c3ccccc3	0.781
35	O=C1c2c(C(=CN1C)c3ccccc3)cc[nH]2	0.715
36	Cc1cc(Nc2cc(ncn2)-c3ccccc3)no1	0.771
37	O=C(N)c1ccnc(-c2ccccc2)c1	0.757
38	O=C(Nc1cc(-c2ccccc2)ccn1)COC	0.748
39	Fc1ccc(NC(=O)C)cc1-c2ccccc2	0.705
40	O=C(Nc1cc(ncn1)-c2ccccc2)C3CC3	0.753
41	O=C(Nc1cc(-c2ccccc2)ccn1)CCC	0.752
42	O=C(Nc1cc(cc(c1)-c2ccccc2)C([O-])=O)C	0.682
43	O=S(=O)(Nc1c(O)ccc(c1)-c2ccccc2)C	0.71
44	n1ccc(-c2c[nH]nn2)cc1-c3ccccc3	0.764
45	O=C(Nc1c(ccc(-c2ccccc2)c1)C)CC	0.713
46	Oc1c(c(nc2ccncc12)-c3ccccc3)C#N	0.765
47	Oc1ccc(cc1C(=O)NC)-c2ccccc2	0.73
48	O=C1C=C(OC)C(=O)c2c(cn(c12)C)-c3ccccc3	0.722
49	O=C1C(=CC(=NN1C)c2ccccc2)C	0.691
50	O=C(Nc1ccc(c(c1)-c2ccccc2)CC)C	0.735
